# Short days, long memories: plastid-to-nucleus signaling links photoperiod to heritable epigenetic change

**DOI:** 10.1093/plphys/kiag183

**Published:** 2026-04-07

**Authors:** Neeta Lohani

**Affiliations:** Assistant Features Editor, Plant Physiology, American Society of Plant Biologists, Rockville, MD 20855-2768, United States; Department of Biotechnology, Thapar Institute for Engineering and Technology, Patiala, Punjab 147004, India

Climate variability and seasonal fluctuations in daylength, temperature, and pathogen pressure require plants to continuously adjust their balance between growth and defense. Epigenetic modifications such as DNA methylation and chromatin remodeling play a central role in this adjustment because they can translate environmental signals into heritable changes in gene expression that persist across generations (Zhang et al. 2018; Cao and Chen 2024). While plastid-to-nucleus retrograde signaling has long been studied in the context of photosynthesis gene regulation and chloroplast biogenesis (Loudya et al. 2024), a separate class of plastids may play a more direct role in environmental sensing. These have been described as “sensory plastids,” and they are found in the epidermis, vasculature, and meristems. They accumulate stress-response proteins rather than photosynthetic machinery and appear to specialize in detecting environmental cues (Beltrán et al. 2018; Mackenzie and Mullineaux 2022). Disruption of the sensory plastid protein MutS HOMOLOG 1 (MSH1) triggers heritable changes in DNA methylation and gene expression in the nucleus, with effects that vary depending on daylength (Virdi et al. 2015; Yang et al. 2020). It remains to be determined, however, whether other sensory plastid proteins can trigger similar epigenetic effects and how these plastid-derived signals interact with photoperiod to shape heritable epigenetic changes.

Recently in *Plant Physiology*, Jeh et al. (2026) addressed these questions by systematically disrupting 4 proteins that localize to these specialized plastids in Arabidopsis: MSH1, an organellar DNA-binding protein involved in stress response; PsbP DOMAIN-CONTAINING PROTEIN 3 (PPD3), a PSII subunit that also influences meristem growth; CAB UNDEREXPRESSED 1 (CUE1), a phosphoenolpyruvate transporter; and SAL1, a bifunctional nucleotidase/phosphatase involved in RNA metabolism. While *msh1*, *cue1*, and *sal1* were studied as loss-of-function mutants, PPD3 was analyzed through overexpression (*PPD3OX*), which produces a mixed population of dwarf and enlarged plants. The authors first confirmed CUE1 as a sensory plastid protein through fluorescent reporter imaging, detecting the CUE1 signal in root tips, epidermis, vasculature, and reproductive tissues but not in mesophyll chloroplasts. They then combined newly generated whole-genome bisulfite sequencing and RNA-seq data for *cue1* and *sal1*(a 3′(2′),5′-bisphosphate nucleotidase) mutants with previously published methylome and transcriptome datasets for *msh1* and *PsbP DOMAIN-CONTAINING PROTEIN 3 overexpression* (*PPD3OX*), all grown under identical 12-h-daylength conditions (as described below, daylength appears to affect the effects of these mutationss). Using k-means clustering and STRINGdb network analysis on the combined datasets, the authors identified gene networks affected by each perturbation and asked how much these networks overlapped across the four mutants.

The resulting methylome signatures were remarkably consistent with each protein's known biology. Disruption of *msh1* predominantly affected abiotic and biotic stress response pathways, while the dwarf *PPD3OX* plants showed changes in meristem identity and cell cycle networks. The *cue1* mutant showed enrichment for circadian rhythm and light-sensing pathways, and *sal1* impacted RNA processing and metabolism. Despite these differences, all 4 mutants converged on shared downstream pathways, including RNA-directed DNA methylation (RdDM) components, translation, and developmental processes. This convergence suggests that diverse sensory plastid signals, though arising from distinct protein perturbations, funnel through a common nuclear epigenetic machinery.

What might serve as the nuclear relay for these plastid-derived signals? The authors focused on the chromatin remodeler HISTONE DEACETYLASE 6 (HDA6) as a candidate nuclear mediator, since the *msh1/hda6* double mutant was previously shown to be lethal specifically at 12-h daylength though viable at 16-h daylength (Yang et al. 2020), hinting at a functional connection. When they examined the *hda6* mutant methylome at 12-h daylength, they identified 2,949 CHG differentially methylated regions, of which 2,862 (97%) fell within gene bodies. Strikingly, 87% of these genes overlapped with differentially methylated genes from at least 1 sensory plastid mutant. The hub genes identified within these CHG regions included important regulators such as TARGET OF RAPAMYCIN (TOR), BRAHMA (BRM), SPLAYED (SYD), and multiple RdDM components. Of the 262 core hub genes in the *hda6* dataset, 162 were also core hubs in at least 1 sensory plastid mutant condition, revealing substantial overlap between the nuclear effects of HDA6 loss and sensory plastid perturbation.

Genetic crosses provided further evidence for this connection. All 4 sensory plastid mutants showed epistatic interactions with *hda6*, and these interactions depended on daylength. The *msh1/hda6* and *sal1/hda6* double mutants were completely inviable at 12-h photoperiod due to gametophytic incompatibility, while *ppd3/hda6* showed abnormal floral development and aerial rosettes at 12-h but not 16-h daylength. Interestingly, the *cue1/hda6* double mutant displayed its most severe phenotype at the 16-h daylength, consistent with a distinct role for CUE1 in light sensing. The authors confirmed the plastid origin of the *msh1/hda6* incompatibility using lines in which MSH1 function was selectively restored to either mitochondria or plastids, demonstrating that depleting plastid MSH1, but not mitochondrial MSH1, led to a significant deficit of double mutant progeny.

The daylength dependence of these genetic interactions raised an important question about the methylome itself. When the authors compared *hda6* methylomes at 12-h versus 16-h daylength, the CHG hypermethylation islands prominent at 12 h were absent at 16 h, and transposable element hypomethylation was more pronounced under long days. Sensory plastid mutants similarly showed milder methylome and phenotype effects at 16 h, with stress-response gene networks prominent at 12 h but lacking at 16 h. Notably, the total number of differentially methylated genes was comparable across photoperiods, indicating that daylength does not broadly alter the scale of methylome repatterning. Instead, photoperiod redirects which genes and networks are targeted, with environmental stress pathways consistently overrepresented under short-day conditions across all mutants tested.

These observations prompted the authors to ask whether *hda6* carries a transgenerational memory of daylength. By growing *hda6* plants through multiple generations under alternating photoperiods, they demonstrated that the 12-h CHG methylome signature required at least 2 generations to become fully established or erased. Passage through a heterozygous state could remove the 12-h memory, and once erased, 2 generations of 12-h growth were needed to reestablish the signature. To connect this memory to the *msh1/hda6* lethality, the authors grew double mutants at 16 h for 2 generations and then shifted them to 12 h. First-generation 12-h *msh1/hda6* plants showed only partial reestablishment of CHG hypermethylation, while second-generation plants acquired additional transposable element methylation and failed to produce seeds. This progressive methylome repatterning directly linked the accumulation of 12-h-specific methylation marks to reproductive incompatibility.

If sensory plastid perturbations and *hda6* converge on stress-response pathways in the methylome, do these epigenetic changes translate into actual stress tolerance? The authors tested this by subjecting plants to sustained heat stress (28 °C) at 12-h daylength. Both *hda6* and *msh1/hda6* plants flowered significantly later than wild type, reflecting enhanced thermotolerance, with the double mutant showing the strongest effect. Critically, F_3_  *hda6* plants that lacked the 12-h methylome memory showed no enhanced heat tolerance, confirming that the 12-h CHG hypermethylation state preconditions the plant for stress resilience. Methylome analysis under heat revealed that *hda6* plants underwent preferential CHG demethylation at the very gene body regions hypermethylated under 12-h conditions, suggesting that this preexisting methylation provides a substrate for stress-responsive chromatin remodeling. For biotic stress, inoculation with the powdery mildew *Golovinomyces cichoracearum* at 12-h daylength showed that *hda6* and *msh1* individually exhibited enhanced resistance, while *msh1/hda6* showed the most effective defense. At 16-h daylength, none of these genotypes differed significantly from wild type, reinforcing the daylength specificity of the response.

The resulting model positions HDA6 as a nuclear gatekeeper that integrates sensory plastid retrograde signals with the circadian clock to calibrate the growth-versus-defense tradeoff in response to photoperiod. HDA6 directly interacts with circadian clock components CCA1, LHY, and TOC1 (Hung et al. 2018; Hung et al. 2019), and the authors propose that under short-day conditions, sensory plastid signals converge through HDA6 to establish CHG methylation marks at stress-response loci, priming the plant for defense (Fig. 1). Several open questions remain, however. The overlap between publicly available HDA6 ChIP-seq peaks and the 12-h CHG differentially methylated regions was modest (8.7%, *P* = 0.0861), though the ChIP-seq data were generated under different photoperiod and tissue conditions; future matched experiments would help clarify this relationship. The identity of the retrograde signal(s) also remains undefined, with reactive oxygen species, calcium, nitric oxide, and the SAL1-dependent phosphoadenosine phosphate retrograde pathway all representing plausible candidates. The powdery mildew data, while compelling in showing *msh1/hda6* as the most resistant genotype, relied on 4 plants per genotype and would benefit from larger-scale replication.

**Figure 1:**
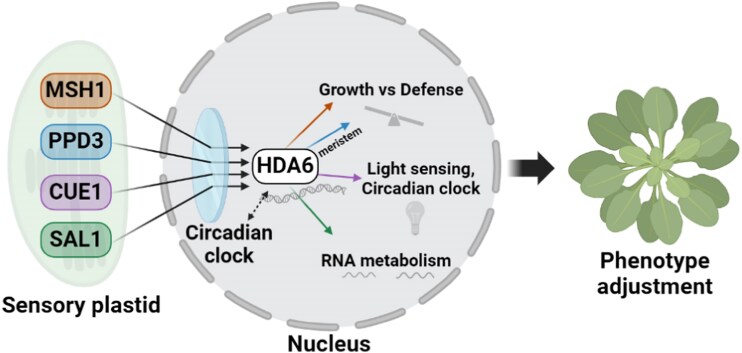
Proposed model of sensory plastid-to-nucleus signaling during environmental sensing. Perturbation of the sensory plastid proteins MSH1, PPD3, CUE1, and SAL1 triggers retrograde signals that converge on HDA6 in the nucleus. HDA6 interacts with circadian clock components to mediate daylength-dependent epigenetic programs that calibrate growth versus defense, light sensing, and RNA metabolism, ultimately adjusting plant phenotype in response to photoperiod. (Jeh et al., Figure 7D).

This study delivers 2 important messages. First, the dramatic differences in methylome landscapes between 12-h and 16-h photoperiods remind us that growth chamber light settings, a variable often treated as routine, can profoundly shape epigenetic readouts and should be carefully reported in future epigenomic studies. Second, by demonstrating that sensory plastid perturbations and HDA6 disruption converge to enhance both abiotic and biotic stress tolerance in a daylength-dependent manner, Jeh et al. provide a compelling framework for understanding how plants write seasonal information into heritable epigenetic marks. As climate change alters seasonal patterns worldwide, investigating whether similar plastid-to-chromatin signaling operates in crop species could open new avenues for leveraging epigenetic variation in breeding programs for climate resilience.

## Recent research articles in *Plant Physiology*:

- Zhou et al. (2024) showed that SANT domain-containing proteins form a complex with HDA6 to coordinate histone acetylation and 2-hydroxyisobutyrylation levels and that disruption of this complex compromises plant heat tolerance in Arabidopsis.
- Hsieh et al. (2024) dissected the multilayered epigenomic roles of HDA6 and the histone demethylases LDL1/LDL2, revealing that these factors act both synergistically and antagonistically to regulate transposable element silencing through coordinated changes in histone acetylation, H3K4 methylation, and DNA methylation in Arabidopsis.

## Data Availability

No new data were generated or analyzed in support of this article.
